# Associations between serum 25– hydroxyvitamin D concentrations and assisted reproductive technology outcomes in women with polycystic ovary syndrome: a cohort study

**DOI:** 10.3389/fendo.2025.1639137

**Published:** 2025-10-15

**Authors:** Li Guo, Xianhua Zheng, Chunming Gu, Zhili Zhang, Xiaodan Zhang, Xiaolin Ruan, Hongyu Li, Qiongdan Mai, Weixiang Wu, Mingyong Luo

**Affiliations:** ^1^ Department of Clinical Laboratory, Guangdong Women and Children Hospital, Guangzhou, China; ^2^ Women and Children’s Hospital, Southern University of Science and Technology, Guangzhou, China

**Keywords:** polycystic ovary syndrome, assisted reproductive technology, vitamin D, pregnancy, live birth

## Abstract

**Background:**

The impact of serum vitamin D levels on the success of assisted reproductive technology (ART) among women with polycystic ovary syndrome (PCOS) has not been fully explored.

**Methods:**

This cohort study enrolled 111 patients with PCOS who underwent *in vitro* fertilization (IVF) or intracyto–plasmic sperm injection (ICSI) treatment at Guangdong Women and Children Hospital from January 2022 to October 2024. Serum 25–hydroxyvitamin D [25(OH)D] concentrations were measured before embryo transfer (ET). The outcomes included the pregnancy rate, live birth rate, and early miscarriage rate. Multivariable logistic regression and the receiver operating characteristic (ROC) curve analyses were performed to evaluate predictive thresholds.

**Results:**

Patients achieving clinical pregnancy (n=76, 68.47%) exhibited significantly higher pre–ET 25(OH)D levels (65.28 ± 18.90 vs. 52.22 ± 15.14, nmol/L, *P* < 0.001) and luteinizing hormone (LH) levels (9.52 ± 6.37 vs. 6.94 ± 4.52, IU/L, *P* = 0.021) compared to non–pregnant counterparts. Logistic regression, after adjusting for LH, demographics and hormonal profiles, revealed that 25(OH)D was significantly associated with pregnancy (B = 0.13, *P* = 0.030). ROC analysis identified 67.95 nmol/L as the optimal 25(OH)D cutoff for predicting pregnancy (with an area under the curve of 0.703, a specificity of 94.3%, and a sensitivity of 36.8%). Furthermore, pregnancies with pre–ET 25(OH)D ≥67.95 nmol/L had significantly higher live birth rates (75.00% vs. 52.08%, *P* = 0.049). Live birth cases maintained higher 25(OH)D levels than early miscarriages (67.63 ± 19.32 vs. 58.22 ± 16.01, nmol/L, *P* = 0.042).

**Conclusions:**

Pre–ET serum vitamin D levels may serve as a modifiable biomarker for optimizing ART success in women with PCOS, associated with enhanced pregnancy likelihood and live birth outcomes. Systematic vitamin D supplementation prior to ET warrants further investigation as a potential adjuvant strategy.

**Clinical Trial Registration:**

https://www.medicalresearch.org.cn, identifier MR-44-25-016940.

## Introduction

Polycystic ovary syndrome (PCOS) constitutes one of the most commonly encountered endocrine and metabolic disorders in reproductive–aged women, with estimated prevalence rates ranging between 10% and 13% (1). The syndrome manifests through heterogeneous clinical features, typically including hyperandrogenemia, ovulatory dysfunction and polycystic ovarian morphology, and is frequently complicated by obesity, insulin resistance, and elevated cardiovascular risk. While the precise pathophysiology remains incompletely understood, current evidence supports a multifactorial etiology involving genetic predisposition, environmental influences, and lifestyle factors (2).

Reproductive impairment, affecting 70–80% of patients with POCS (3), is largely attributed to endocrine dysregulation characterized by reduced follicle–stimulating hormone (FSH) levels impairing follicular maturation, coupled with elevated luteinizing hormone (LH) and androgen concentrations. This hormonal imbalance disrupts the menstrual cycle, leading to oligo/anovulation and subsequent infertility. For patients who fail to conceive through lifestyle adjustments and ovulation induction therapy, assisted reproductive technology (ART)–particularly *in vitro* fertilization (IVF) and intracytoplasmic sperm injection (ICSI)–has become essential for achieving conception. These techniques assist conception through a series of complex procedures, including ovarian stimulation with medication, *in vitro* fertilization, and embryo transfer (ET). However, treatment outcomes remain compromised due to PCOS–related pathophysiological complexities, with lower–than–anticipated pregnancy rates and heightened risks of both procedural complications and adverse gestational outcomes (4–6). These challenges underscore the need for systematic exploration of adjustable prognostic determinants to optimize therapeutic strategies.

Accumulating evidence indicates that vitamin D plays a crucial role in human reproductive health. The ubiquitous distribution of vitamin D receptors in female reproductive tissues underpins their regulatory roles in ovarian steroidogenesis, folliculogenesis, endometrial receptivity maturation, and embryo implantation, thereby influencing critical reproductive phases from menstrual cyclicity to fertility outcomes (7–9). Notably, PCOS populations exhibit higher prevalence of vitamin D deficiency compared to the general population (10, 11). Emerging evidence links this deficiency to suboptimal ART outcomes. Some studies indicate that maintaining an adequate level of vitamin D might be helpful in improving the pregnancy rate and live birth rate of PCOS populations after undergoing ART (7, 12, 13). Nevertheless, current evidence remains limited regarding vitamin D’s specific impact on ART outcomes in PCOS populations.

This study is designed to investigate the association between serum vitamin D status and pregnancy outcomes of ART in PCOS populations. Our findings may enhance understanding of vitamin D’s therapeutic potential in PCOS–related infertility management and inform strategies for optimizing ART protocols.

## Methods

### Study design

This prospective cohort study investigated the association between pre-embryo transfer serum 25-hydroxyvitamin D [25(OH)D] concentrations and IVF/ICSI outcomes in women with PCOS. Consecutively enrolled PCOS patients undergoing fresh or frozen-thawed embryo transfer cycles at Guangdong Women and Children Hospital (January 2022–October 2024) were included. This study was approved by the Ethics Committee of Guangdong Women and Children Hospital (Approval No. 20251009) and conducted in accordance with the ethical principles of the Declaration of Helsinki.

### Patients

We collected data of patients with PCOS who received IVF/ICSI treatment at Guangdong Women and Children Hospital from January 2022 to October 2024.

#### Inclusion criteria

(1) Meeting the Rotterdam diagnostic criteria: PCOS is diagnosed if two of the following three conditions are satisfied: hyperandrogenism, oligoovulation, and polycystic ovarian changes as assessed by ultrasound (>12 cysts or ovarian volume >10 mL); (2) aged 18–45 years; and (3) receiving IVF/ICSI treatment at Guangdong Women and Children Hospital.

#### Exclusion criteria

(1) Individuals with systemic diseases such as metabolic syndrome, diabetes, hyperlipidemia, and cardiovascular diseases; (2) patients with thyroid diseases or congenital adrenal hyperplasia; (3) patients with severe endometriosis, ovarian or cervical tumors, or HPV infection; (4) patients who smoke and/or consume alcohol; (5) patients who use any drugs that affect reproductive physiology within three months before the study, except for the treatment of PCOS; (6) patients who employ any medications that have an impact on reproductive physiology within three months preceding the study, with the exception of those for the treatment of PCOS; (7) patients with any autoimmune diseases; and (8) patients undergoing ART due to male factor infertility.

### Clinical data

The clinical data of patients, including age, body mass index (BMI), sex hormone–binding globulin (SHGB), and hormone levels, including serum follicle–stimulating hormone (FSH), luteinizing hormone (LH), prolactin (PRL), testosterone, anti–Müllerian hormone (AMH), and androgens, were collected.

### Measurement of the 25(OH)D concentration

The acquisition of blood samples during the IVF/ICSI treatment of patients for the purpose of evaluating vitamin D levels. All the vitamin D assays were carried out punctually in our clinical laboratory. Serum vitamin D was determined by chemiluminescence technology to measure total 25–hydroxyvitamin D [25(OH)D] (Architect, Abbott Diagnostics, USA). Its detection sensitivity is 8.8 nmol/L, and the inter–assay and intra–assay coefficients of variation are 9.0% and 5.5%, respectively.

### IVF/ICSI–ET procedure and outcomes

The IVF/ICSI–ET procedure and its outcomes are referred to in the published literature by Xu et al. (14) Clinical pregnancy was defined as positive serum human chorionic gonadotropin (hCG) and the presence of a gestational sac and fetal heart activity on ultrasound examination. The primary outcome was pregnancy, and the secondary outcomes included live birth and early miscarriage. Early miscarriage refers to miscarriage that occurs within 12 weeks of gestation.

### Statistical analysis

All the data were statistically analyzed via SPSS 24.0, and *P* < 0.05 was considered statistically significant. Continuous variables with a normal distribution are expressed as the mean ± SD, and Student’s t test was used to compare the differences in patient characteristics between groups. Data with a non-normal distribution are represented by the median and confidence interval, and the Mann–Whitney U test was used. Categorical variables are expressed as numbers and percentages, and the Pearson chi–square test or Fisher’s exact test was used for comparisons. Univariate logistic regression was used to calculate the OR for the preliminary test of the association between VD levels and IVF/ICSI treatment outcomes. Multivariate logistic regression was used to adjust the OR estimates of relevant covariates. For the primary outcome of treatment, pregnancy, we created two models: Model 1 included factors with significant differences in univariate logistic regression, namely, LH; Model 2 included adjusted Model 1, age, BMI, vitamin D supplement medications, SHBG, testosterone, AMH, PRL, LH, FSH, androstenedione, and the homeostasis model assessment insulin resistance index (HOMA–IR).

## Results

### General characteristics of PCOS patients undergoing ART

A total of 111 patients with PCOS who were undergoing IVF/ICSI treatment were included in this study. Prior to ET, tests for serum 25(OH) D and other hormone levels were carried out. The details of the general characteristics, including demographics and laboratory data, are summarized in **Table 1**. In accordance with the clinical pregnancy diagnostic criteria, these patients were divided into two groups: the gravidity group (with a mean age of 30.64 ± 3.95 years, a mean BMI of 22.34 ± 3.14, and a total of 76 patients, accounting for 68.47%) and the nonpregnancy group (with a mean age of 30.60 ± 4.10 years, a mean BMI of 23.01 ± 4.00, and a total of 35 patients, accounting for 31.53%). There was no significant difference between the two groups with respect to the use of vitamin D supplement medications before ET (*P* = 0.906). Compared with those in non-pregnant patients, 25(OH)D (65.28 ± 18.90 vs. 52.22 ± 15.14, nmol/L, *P* < 0.001) and LH (9.52 ± 6.37 vs. 6.94 ± 4.52, IU/L, *P* = 0.021) levels in pregnant patients were significantly elevated. Other clinical indicators, such as SHBG (73.48 ± 58.69 vs. 57.85 ± 44.17, nmol/L, *P* = 0.312), testosterone (1.40 ± 7.29 vs. 0.47 ± 0.61, ng/mL, *P* = 0.535), AMH (10.24 ± 8.32 vs. 7.95 ± 4.58, ng/mL, *P* = 0.167), PRL (27.16 ± 62.12 vs. 24.95 ± 22.36, ng/mL, *P* = 0.862), FSH (6.01 ± 1.82 vs. 6.06 ± 2.21, IU/L, *P* = 0.896), androstenedione (10.06 ± 4.97 vs. 9.10 ± 4.75, nmol/L, *P* = 0.958), hemoglobin A1c (5.17 ± 0.53 vs. 5.23 ± 0.34, %, *P* = 0.668) and HOMA–IR (1.98 ± 2.21 vs. 2.13 ± 1.26, *P* = 0.706) levels were did not exhibit significant differences.

**Table 1:** Demographic features of PCOS patients who have undergone IVF/ICSI.

Variables	Gravidity (n=76)	Nonpregnancy (n=35)	*P*
Age(year)	30.64 ± 3.95	30.60 ± 4.10	0.956
BMI	22.34 ± 3.14	23.01 ± 4.00	0.351
Vitamin D supplement medications (%)	36(47.37%)	17(48.57%)	0.906
serum 25(OH)D level (nmol/L)	65.28 ± 18.90	52.22 ± 15.14	**<0.001**
25(OH)D level <50nmol/L (n, %)	13(17.11%)	14(40.00%)	
25(OH)D level ≥50, <75nmol/L (n, %)	45(59.21%)	19(54.29%)	
25(OH)D level ≥75nmol/L (n, %)	18(23.68%)	2(5.71%)	
SHBG (nmol/L)	73.48 ± 58.69	57.85 ± 44.17	0.312
Testosterone (ng/mL)	1.40 ± 7.29	0.47 ± 0.61	0.535
AMH (ng/mL)	10.24 ± 8.32	7.95 ± 4.58	0.167
PRL (ng/mL)	27.16 ± 62.12	24.95 ± 22.36	0.862
LH (IU/L)	9.52 ± 6.37	6.94 ± 4.52	**0.021**
FSH (IU/L)	6.01 ± 1.82	6.06 ± 2.21	0.896
Androstenedione (nmol/L)	10.06 ± 4.97	9.10 ± 4.75	0.958
Hemoglobin A1c (%)	5.17 ± 0.53	5.23 ± 0.34	0.668
HOMA–IR	1.98 ± 2.21	2.13 ± 1.26	0.706

Bold values indicate statistical significance at P < 0.05.

### Associations between vitamin D and gravidity in PCOS patients who had undergone ART

Univariate logistic regression analysis revealed that the serum 25(OH)D (B = 0.05, *P* = 0.001) and LH (B = 0.09, *P* = 0.050) levels were strongly correlated with pregnancy in PCOS patients who had undergone IVF/ICSI (**Table 2**).

**Table 2:** Univariate analysis of factors associated with gravidity in participants with PCOS.

Gravidity (+)	B, SE (n=76)	Odds ratio (95% CI)	*P*
Age(year)	0.00, 0.05	1.00(0.91–1.11)	0.956
BMI	–0.06,0.06	0.94(0.84–1.06)	0.348
Vitamin D supplement medications (%)	–0.05,0.41	0.95(0.43–2.12)	0.906
serum 25(OH)D level (nmol/L)	0.05,0.02	1.05(1.02–1.08)	**0.001**
SHBG (nmol/L)	0.01,0.01	1.01(0.99–1.02)	0.313
Testosterone (ng/mL)	0.22,0.52	1.24(0.45–3.46)	0.680
AMH (ng/mL)	0.06,0.05	1.06(0.98–1.16)	0.165
PRL (ng/mL)	0.00,0.01	1.00(0.99–1.01)	0.861
LH (IU/L)	0.09,0.05	1.09(1.00–1.20)	**0.050**
FSH (IU/L)	–0.02,0.11	0.99(0.79–1.23)	0.895
Androstenedione (nmol/L)	0.04,0.06	1.04(0.93–1.17)	0.465
Hemoglobin A1c (%)	–0.25,0.57	0.78 (0.26–2.38)	0.664
HOMA–IR	–0.04,0.10	0.96(0.79–1.17)	0.705

Bold values indicate statistical significance at P < 0.05.

The relationship between vitamin D and gravidity in PCOS patients who had undergone ART was evaluated via a multivariate logistic regression model. After adjusting for LH, it was revealed that 25(OH)D levels were significantly linked with pregnancy (Model 1, *P* = 0.003). Similar results were obtained after further adjustment for age, BMI, history of taking vitamin D supplement medications, SHBG, testosterone, AMH, PRL, FSH, androstenedione and HOMA–IR (Model 2, *P* = 0.030) (**Table 3**).

**Table 3:** Association between serum vitamin D and gravidity in participants with PCOS.

Gravidity (+)	B, SE(n=76)	Odds ratio (95% CI)	*P*
25(OH)D level (nmol/L)			
Model 1	0.05,0.02	1.05(1.02–1.09)	**0.003**
Model 2	0.13,0.06	1.14 (1.01–1.29)	**0.030**

CI, confidence interval; SE, standard error

a Multiple factor logistic regression Model, Model 1: adjusted for LH; Model 2: adjusted for Model 1 and age, BMI and vitamin D supplement medications, SHBG, testosterone AMH, PRL, LH, FSH, androstenedione and HOMA–IR.

Bold values indicate statistical significance at P < 0.05.

**Figure 1:**
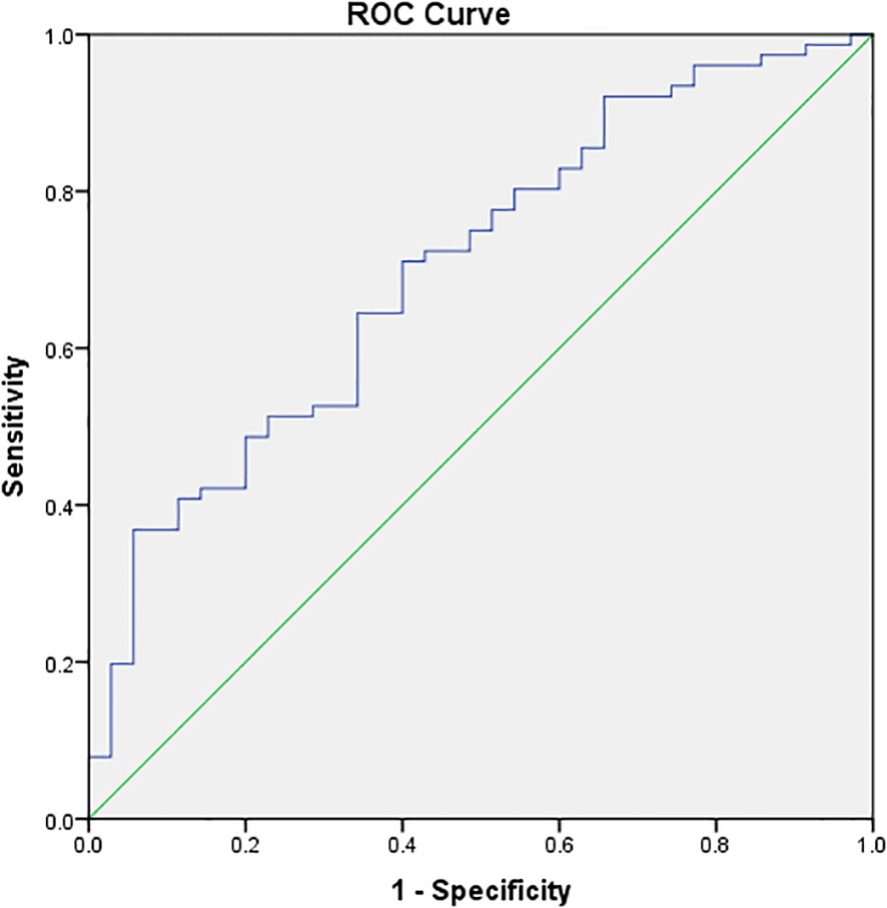
ROC curve model analysis of vitamin D and pregnancy. The X–axis represents 1–specificity, and the Y–axis represents sensitivity. The cutoff point of vitamin D for predicting pregnancy (Youden’s index) was 67.95 nmol/L, which was the best predictive value (area under the curve: 0.703, specificity: 94.3% and sensitivity: 36.8%).

In accordance with the analysis of the receiver operating characteristic (ROC) curve model, the cutoff point of serum 25(OH)D levels for predicting whether PCOS patients could achieve pregnancy subsequent to ART (Youden’s index) was 67.95 nmol/L, which demonstrated the optimal predictive effect (area under the curve: 0.703; specificity: 94.3%; sensitivity: 36.8%) (**Figure 1**).

### Association between live birth and vitamin D in pregnant patients after receiving ART

To further investigate the relationship between vitamin D levels and pregnancy outcomes in patients with PCOS who underwent IVF/ICSI, we subdivided the pregnant patients into two subgroups according to the optimal predicted 25(OH) D levels (**Table 4**). Patients with 25(OH)D ≥ 67.95 nmol/L were more prone to live birth after a successful pregnancy (75.00% vs. 52.08%, *P* = 0.049).

**Table 4:** Live birth rate after pregnancy in PCOS patients with high serum vitamin D levels ^a^.

	Vitamin D <67.95 nmol/L (n=48)	Vitamin D ≥67.95 nmol/L (n=28)	*P*
Live birth (%)	25(52.08%)	21(75.00%)	**0.049**

a Values are patient numbers (%) or medians and ranges.

Bold values indicate statistical significance at P < 0.05.

We subsequently compared the levels of vitamin D and other hormones between live births and early miscarriages within the pregnancy group (**Table 5**). The results demonstrated that the serum 25(OH)D levels in patients with live births were markedly greater than those in patients with early miscarriages (67.63 ± 19.32 vs. 58.22 ± 16.01, nmol/L, *P* = 0.042), whereas the other indicators were not significantly different.

**Table 5:** Demographic features of PCOS patients after IVF with different pregnancy outcomes.

Variables	Live birth (n=46)	Early miscarriage (n=25)	*P*
Age (year)	30.39 ± 4.03	30.96 ± 4.20	0.577
BMI	22.46 ± 3.28	22.42 ± 2.91	0.955
Vitamin D supplement medications (%)	21(47.37%)	14(48.57%)	0.316
serum 25(OH)D level (nmol/L)	67.63 ± 19.32	58.22 ± 16.01	**0.042**
SHBG (nmol/L)	87.03 ± 71.33	62.03 ± 34.17	0.181
Testosterone (ng/mL)	2.06 ± 9.37	0.42 ± 0.30	0.406
AMH (ng/mL)	10.33 ± 9.83	10.53 ± 5.37	0.927
PRL (ng/mL)	31.14 ± 79.53	21.90 ± 12.59	0.584
LH (IU/L)	9.31 ± 7.11	9.28 ± 4.61	0.982
FSH (IU/L)	6.01 ± 1.82	6.06 ± 2.21	0.409
Androstenedione (nmol/L)	9.75 ± 4.89	10.55 ± 4.61	0.606
Hemoglobin A1c (%)	5.14 ± 0.41	5.09 ± 0.22	0.652
HOMA–IR	1.76 ± 1.12	1.87 ± 1.45	0.728

Bold values indicate statistical significance at P < 0.05.

## Discussion

In the present study, we investigated the relationship between serum vitamin D levels and the therapeutic outcomes of ART in patients with PCOS. The results revealed that there was a positive correlation between vitamin D levels and the pregnancy rate of ART in PCOS patients. Moreover, among the PCOS patients who successfully conceived, those with high vitamin D levels presented a higher live birth rate, and the serum vitamin D level of the live birth patients was clearly higher than that of the patients with early miscarriage.

Vitamin D is a fat–soluble steroid hormone, and its receptors exist in various tissues. In addition to playing crucial roles in aspects such as bone health, cardiovascular health, and immune function, its function in female reproductive health has also drawn increasing attention. Research has revealed that vitamin D participates in the follicular development process and the proliferation process of granulosa cells through mechanisms such as regulating hormone levels and anti–inflammatory effects (8, 15). Furthermore, vitamin D is also involved in embryo implantation and the development of the endometrium; therefore vitamin D influences the diverse reproductive cycles of women (16).

Vitamin D deficiency is a risk factor for multiple gynecological and obstetrical diseases, including PCOS and infertility (15, 17). Clinical investigations have shown that in women with PCOS, vitamin D deficiency is related to the occurrence of hyperandrogenism, insulin resistance, metabolic and endocrine disorders, as well as oxidative stress and a proinflammatory environment (11, 18, 19). Nevertheless, some studies have revealed that supplementation with vitamin D has no beneficial effect on patients with PCOS (20, 21). In terms of PCOS–related pregnancies, in contrast to PCOS women with a normal vitamin D level who require ovulation induction, PCOS women with vitamin D deficiency exhibit a lower ovulation rate and live birth rate and an increased risk of early miscarriage (11). Moreover, supplementation with vitamin D can increase the ovulation rate in PCOS patients (22). However, at present, studies on the impact of vitamin D on IVF outcomes in PCOS patients are relatively rare. Hence, more studies are needed for further in–depth exploration. In this study, after excluding the influences of age, BMI, vitamin D supplementation and other hormones, we discovered that the serum vitamin D levels of PCOS patients undergoing IVF were significantly positively correlated with the pregnancy rate after treatment.

Currently, there remains controversy regarding the impact of vitamin D on the treatment outcomes of ART. The majority of studies have shown that maintaining an adequate level of vitamin D leads to a higher pregnancy rate and live birth rate after assisted reproductive treatment (7, 12). However, some studies suggest that the serum vitamin D level does not affect the treatment results of ART, including the pregnancy rate, live birth rate, and miscarriage rate (23). Some studies have indicated that there is a nonlinear correlation between vitamin D and ART results. Specifically, when the vitamin D level is low, it does not affect the ART results, and when an adequate vitamin D level is maintained during ART, the pregnancy rate and live birth rate tend to be higher. Researchers posit that this might be due to the threshold effect of the vitamin D concentration, which leads to inconsistent research results. Xu et al. proposed that 24 ng/ml (approximately 60 nM/L) might be a suitable threshold for predicting reproductive outcomes (7). In our study, we found that a vitamin D level exceeding 67.95 nmol/L before embryo transfer in PCOS patients was the best predictor of pregnancy and live birth. Compared with that of patients who ultimately experienced early miscarriage, the vitamin D level of live–birth patients was markedly greater.

Luteinizing hormone, a gonadotropin secreted by the anterior pituitary gland, stimulates follicular cells to produce androgens and promotes follicle maturation and granulosa cell ovulation (24). In ART, the administration of exogenous recombinant LH has been widely utilized to stimulate follicle development. Previous investigations have demonstrated that supplementing LH is helpful for improving oocyte quality, follicle growth, and pregnancy outcomes (25, 26). In this study, we observed that the LH level in the pregnant group was significantly greater than that in the non-pregnant group, which might be a consequence of the influence of ovulation induction drugs. These findings suggest that reasonable regulation of LH levels during the ART treatment of PCOS patients is beneficial for improving treatment outcomes.

Despite the fact that this study has provided valuable insights, there are nevertheless certain limitations.

First, this is a single–center study that only incorporates Chinese individuals and has a relatively restricted sample size, which might impact the universality of the results. Future multicenter studies with larger cohorts are needed to validate our findings.

Second, we were unable to completely rule out all potential confounding factors. Specifically, data on vitamin D supplementation, nutritional status, and sunlight exposure—key factors known to influence serum vitamin D concentrations —were not systematically collected (19, 27). Seasonal variations in vitamin D levels, a well-documented phenomenon (27), were also not accounted for in the analysis.

Third, although couples with known male infertility factors were excluded from this study, we did not evaluate subtle male parameters (e.g., sperm DNA fragmentation) or partners’ vitamin D status. Research indicates that vitamin D plays a critical role in maintaining male reproductive health by influencing spermatogenesis, sperm motility, and hormonal regulation (16, 28, 29).

Fourth, in ART, embryo transfer occurs at two key stages: cleavage-stage transfer and blastocyst-stage transfer. Frozen embryo transfer (FET) is increasingly preferred for achieving pregnancy post-IVF, particularly benefiting anovulatory women. In this study, the developmental stage of transferred embryos was not specified or analyzed; future research should incorporate this variable (30, 31).

Finally, this was a cross-sectional study with vitamin D measured only pre-transfer; dynamic monitoring of vitamin D fluctuations during treatment was not performed, preventing assessment of its longitudinal impact on outcomes.

These limitations may contribute to the relatively low sensitivity of our predictive model. Future studies should incorporate these variables and employ longitudinal designs to better elucidate the causal relationship between vitamin D dynamics and ART outcomes.

In summary, this study provides a new perspective on the application of vitamin D in ART, suggesting that the serum vitamin D level in PCOS patients may have predictive value for their pregnancy outcomes. These findings might offer new evidence for optimizing reproductive treatment regimens for patients with PCOS.

## Conclusion

In this prospective study of PCOS women undergoing IVF/ICSI treatment, pre-embryo transfer serum 25(OH)D concentrations demonstrated significant predictive utility for ART outcomes. Key findings revealed that higher 25(OH)D levels (≥68 nmol/L) were independently associated with increased clinical pregnancy rates, enhanced live birth probabilities, and reduced early miscarriage risks. The identification of a 25(OH)D threshold at 68 nmol/L—characterized by high specificity (94.3%) for pregnancy prediction—suggests its potential as a clinically actionable biomarker. While the modest sensitivity (36.8%) underscores the multifactorial nature of ART success, our data imply that optimizing vitamin D status prior to embryo transfer may represent a modifiable strategy to improve reproductive outcomes in this population. These findings advocate for systematic screening and targeted supplementation in vitamin D-deficient PCOS patients undergoing ART. Further randomized controlled trials are warranted to establish causality and refine evidence-based thresholds for clinical implementation.

## Data Availability

The raw data supporting the conclusions of this article will be made available by the authors, without undue reservation.
